# Simulation for a Mems-Based CTRNN Ultra-Low Power Implementation of Human Activity Recognition

**DOI:** 10.3389/fdgth.2021.731076

**Published:** 2021-09-22

**Authors:** Muhammad Emad-Ud-Din, Mohammad H. Hasan, Roozbeh Jafari, Siavash Pourkamali, Fadi Alsaleem

**Affiliations:** ^1^Department of Computer Science and Engineering, Texas A&M University, College Station, TX, United States; ^2^Department of Earth and Space Sciences, Columbus State University, Columbus, OH, United States; ^3^Department of Electrical and Computer Engineering, University of Texas at Dallas, Dallas, TX, United States; ^4^Department of Biomedical Engineering, Texas A&M University, College Station, TX, United States; ^5^Department of Electrical and Computer Engineering, Texas A&M University, College Station, TX, United States; ^6^Durham School of Architectural Engineering and Construction, University of Nebraska—Lincoln, Omaha, NE, United States

**Keywords:** MEMS, human activity recognition, LSTM – long short-term memory, continuous time recurrent neural network, recurrent neural networks

## Abstract

This paper presents an energy-efficient classification framework that performs human activity recognition (HAR). Typically, HAR classification tasks require a computational platform that includes a processor and memory along with sensors and their interfaces, all of which consume significant power. The presented framework employs microelectromechanical systems (MEMS) based Continuous Time Recurrent Neural Network (CTRNN) to perform HAR tasks very efficiently. In a real physical implementation, we show that the MEMS-CTRNN nodes can perform computing while consuming power on a nano-watts scale compared to the micro-watts state-of-the-art hardware. We also confirm that this huge power reduction doesn't come at the expense of reduced performance by evaluating its accuracy to classify the highly cited human activity recognition dataset (HAPT). Our simulation results show that the HAR framework that consists of a training module, and a network of MEMS-based CTRNN nodes, provides HAR classification accuracy for the HAPT that is comparable to traditional CTRNN and other Recurrent Neural Network (RNN) implantations. For example, we show that the MEMS-based CTRNN model average accuracy for the worst-case scenario of not using pre-processing techniques, such as quantization, to classify 5 different activities is 77.94% compared to 78.48% using the traditional CTRNN.

## Introduction

Human activity recognition (HAR) presents significant opportunities for various domains, from healthcare applications such as patient monitoring to fitness tracking and productivity assessment, where a system can efficiently detect a specific subject movement, such as standing up or sitting down, while ignoring other activities. For example, in an office space environment, a limited set of activities are important for a productivity assessment application (e.g., typing on a keyboard while seated on a chair may count as work while the remaining activities may not be considered as work). When a person performs an activity, clear, yet complex and distinct, motion signatures are acquired from their body parts for each activity type. Multiple machine learning methods have been proposed to automatically perform HAR by learning those signatures from highly sampled acceleration measurements. Recurrent neural networks (RNNs), in comparison to the typical feedforward neural networks (FFNNs), have been shown to achieve the highest accuracy, as they can process and encode the sequential temporal information contained in the motion data to distinguish a specific day-to-day activity like walking from jumping. Wrist-wearable devices embedded with many highly sensitive and fast-response Microelectromechanical Systems (MEMS) Inertial Measurement Units (IMUs), such as accelerometers and gyroscopes, are among the best candidates for performing this kind of detection. They can also ensure a high degree of adoption because they are perceived as non-intrusive pieces of jewelry. The stringent power requirement and short battery life of wearable devices, however, prevent them from having enough processing power to continuously perform RNN or other kinds of machine learning locally. Moreover, the high energy cost of wirelessly transmitting data, limits the amount of raw sensor data that can be sent and processed externally (1, 2). These limitations reduce the accuracy and applicability of machine learning models, especially when data are sampled at very high rates, which leads to latencies (3).

Very tiny biological systems, such as some insects, have similar constraints and solve such problems by moving some intelligence to the sensor level to efficiently extract complex information (4). Inspired by computing in insects, it is desirable to develop sensor platforms capable of sensing simple inputs and processing them at the sensor level for extracting complex information such as feature extraction or classification, without using microprocessor power. Toward this end, our research group was one of the first to recently develop such a novel solution using a network of mechanically coupled microelectromechanical systems (MEMS) accelerometers that can perform a simple binary classification problem such as distinguishing a square acceleration signal from a triangular one (5). In that work, we have shown that nonlinear detection instability, facilitated by pull-in or nonlinear arch geometries, and hysteresis, enables small MEMS networks to qualitatively capture critical properties of CTRNNs. Hence, allowing MEMS networks to perform time-series computation. However, a significant limitation of our previous work is the lack of a suitable training algorithm for the sensor coupling weights that can train them to perform complex classification applications such as HAR. Instead, a trial-and-error approach was followed to find suitable coupling weights to perform the simple binary classification problem. In this paper, we expand our novel MEMS computing platform by adapting the well-known training algorithms of backpropagation to train the mechanical coupling weight of a network of MEMS accelerometers to perform HAR. We show that this novel architecture enables the MEMS computing network to perform complex classification tasks with comparable accuracy numbers while consuming orders of magnitudes power compared to traditional approaches. The organization of this article is as follows: In Section literature review, contemporary energy-efficient HAR methods are explored and compared. In section Method, we introduce the MEMS-CTRNN node and its integration into the HAR pipeline. We also outline the optimization steps along with the dataset formatting and MEMS-CTRNN architecture details. In section performance evaluation we outline the experimentation and performance analysis approach and results. Finally, in section conclusion we conclude and summarize our findings.

## Literature Review

There have been several attempts in the literature to reduce the algorithm complexity and hence the energy consumption to perform RNN computing. These include a host of optimizations including feature section, quantization, compression, and non-linear approximation (6). For example, various optimization techniques that increase the computational efficiency for the feature selection process have been proposed in the literature (6–9). Techniques like varying the acceleration sampling frequency and window size are also common. Such techniques offer computational power reduction of as much as 44.23% (10, 11) without impacting the classification accuracy. Another technique presented in (12) uses a clustering-center-based pre-classification strategy to reduce the call frequency of the model, thus reducing the overall power consumption by 49%. Techniques like Quantization and Piece-wise Linear Representation (PRL) have been shown to reduce the computing complexity of RNNs. However, such techniques are computationally infeasible when it comes to their implementation on low-power embedded systems (13). Fixed and large sliding window sizes are usually beneficial for power consumption, but the sliding window size is a highly subjective parameter and greatly depends upon the problem and activity characteristics (14).

Optimizing the computer hardware is another direction to reduce the energy consumption to perform RNN computing. In this regard, Table 1 shows different RNN algorithms implementation using state-of-the-art computing platforms. These RNN algorithms include Long Short-Term Memory (LSTM), bidirectional LSTM, fully connected networks like Deep Neural Networks (DNN), and sparsity-aware approximate LSTM. The table compares the algorithms implementation power consumption to perform complex tasks such as speech recognition or image captioning. In all cases, the energy efficiency of each hardware is given in terms of the number of operations (OPS) per watt. Assuming the minimum required of 1.3 TOPS/s to perform RNN calculation (15), the algorithm's energy consumption per second is also estimated in the table. The data in the table shows a minimum reported power consumption of 2.95 m watts using FPGA (16). Assuming an extra 50% power reduction by implementing some of the aforementioned optimization techniques, this might be impractical for ultra-low energy consumption devices such as wearables (1, 2).

**Table 1:** Energy consumption estimation for typical RNN.

**Model (Reference)**	**Platform**	**Efficiency(Giga OPS/watt)**	**Estimated Energy (watt)**	**Application Area(s)**
LSTM (17)	ASIC, CMOS 65 nm	27000	5 mW	Speech Recognition, Image Captioning
Bi-LSTM (16)	FPGA, Zynq−7000 XC7Z045 @ 142 MHz	44	2.95 mW	Image Captioning
GRU (18)	ASIC, CMOS 65 nm @ 400 MHz	2380	54 mW	Image Captioning
DNN (19)	FPGA, Intel Cyclone V @ 250 MHz	5156	25 mW	Image Captioning
Sparsity-aware, approximate-LSTM (20)	CMOS 65 nm	160	33 mW	Speech Recognition

To overcome such challenges, in this paper we present a framework that enables our recent MEMS-based neuromorphic computing approach (5), a bio-inspired computing scheme aiming to implement computing in an analog fashion, to tackle challenging applications while adhering to the stringent energy requirements of wearable devices. It is expected that the physical implementation of the MEMS computing solution presented in this paper consumes orders of magnitudes less power compared to the state of art computing platforms in Table 1. The solution is made of an array of coupled electrostatic MEMS devices, each acting as a neuron in a neural. To estimate the MESM network power consumption, we assume during operation, the switching of each MEMS neuron involves charging a capacitor 0.5 pico-Farad, which is a good approximation for capacitance associated with a typical electrostatic MEMS device. Assuming an operating voltage of 5V and a worst-case scenario switching frequency of 100 Hz, then the required energy to charge up the MEMS capacitance per second is 0.625 n watt. For a MEMS CTRNN with 100 neurons, the total estimated power consumption is only 6.25 n watts. Moreover, compared to baseline, additional energy savings can be harnessed at the system level using this framework with the option of performing some computing at the sensor level, thus reducing the need for sensor-processor communication, memory usage, and conditioning circuit. Finally, it is important to mention that while the power consumption advantages of MEMS CTRNN are clear based on the above simple calculation, the major focus of this paper is to confirm that this MEMS CTRNN has comparable accuracy performance to other RNN implementations.

## Method

RNNs utilize internal memory through self-feedback to preserve the sequences of input data during training (21). Thus, the RNNs have shown great success in sensory applications such as image, video, and audio processing, as well as HAR (22). A special, yet rather a complex form of RNN, known as a Continuous-Time Recurrent Neural Network (CTRNN) (23), uses differential equations to describe the activation level of the neurons (see Equation (1) below).


(1) $$\begin{array}{l} {\dot{y}}_{i}\;=\;f_{i}\left(y_{1},\;\ldots ,\;y_{N}\right)\;=\;\frac{1}{\tau_{i}}\left(-y_{i}+\sum_{j\;=\;1}^{N}w_{ij}\sigma \left(y_{j}\right)\;+\;h_{i}\;+\;I_{i}\right),\\ \;i\;=\;1,2,\ldots ,N\ldots \end{array}$$

where σ is an activation function, which is often sigmoidal, τ_*i*_ and *y*_*i*_ are the time constant and activation level of neuron *I*, respectively, *w*_*ij*_ is the connection strength between the *i*^*th*^ neuron and the *j*^*th*^ neuron, *h* is a bias term, *I*_*i*_ is the input to the *i*^*th*^ neuron, and the dot operator represents the time derivative.

CTRNNs have emerged as an attractive RNN machine learning option as they offer significant dynamical richness while requiring fewer neurons than other RNNs for high-level learning (24). Nevertheless, CTRNNs are seldom implemented because they are computationally expensive for real-time implementation as they require simultaneous solutions of multiple highly coupled differential equations. In previous work, we have shown that the complex computational requirements of implementing CTRNNs may be bypassed by emulating the operation of CTRNNs using physical hardware. Specifically, the dynamics of a small network of electrostatically coupled MEMS cantilevers were used for performing CTRNN computing in an analog fashion that reduces power consumption by multiple orders of magnitudes (5). However, as there is no current literature that addresses the automatic training of this novel technique, the previously demonstrated small MEMS CTRNN was simply trained by trial-and-error for a simple classification task. To address this challenge, in this paper, we present a training framework that can be used to train the novel MEMS-based CTRNN architectures to tackle complex applications. This model will be referred to as MEMS-CTRNN. As a case study, a MEMS-CTRNN will be trained to perform HAR.

**Figure 1:**
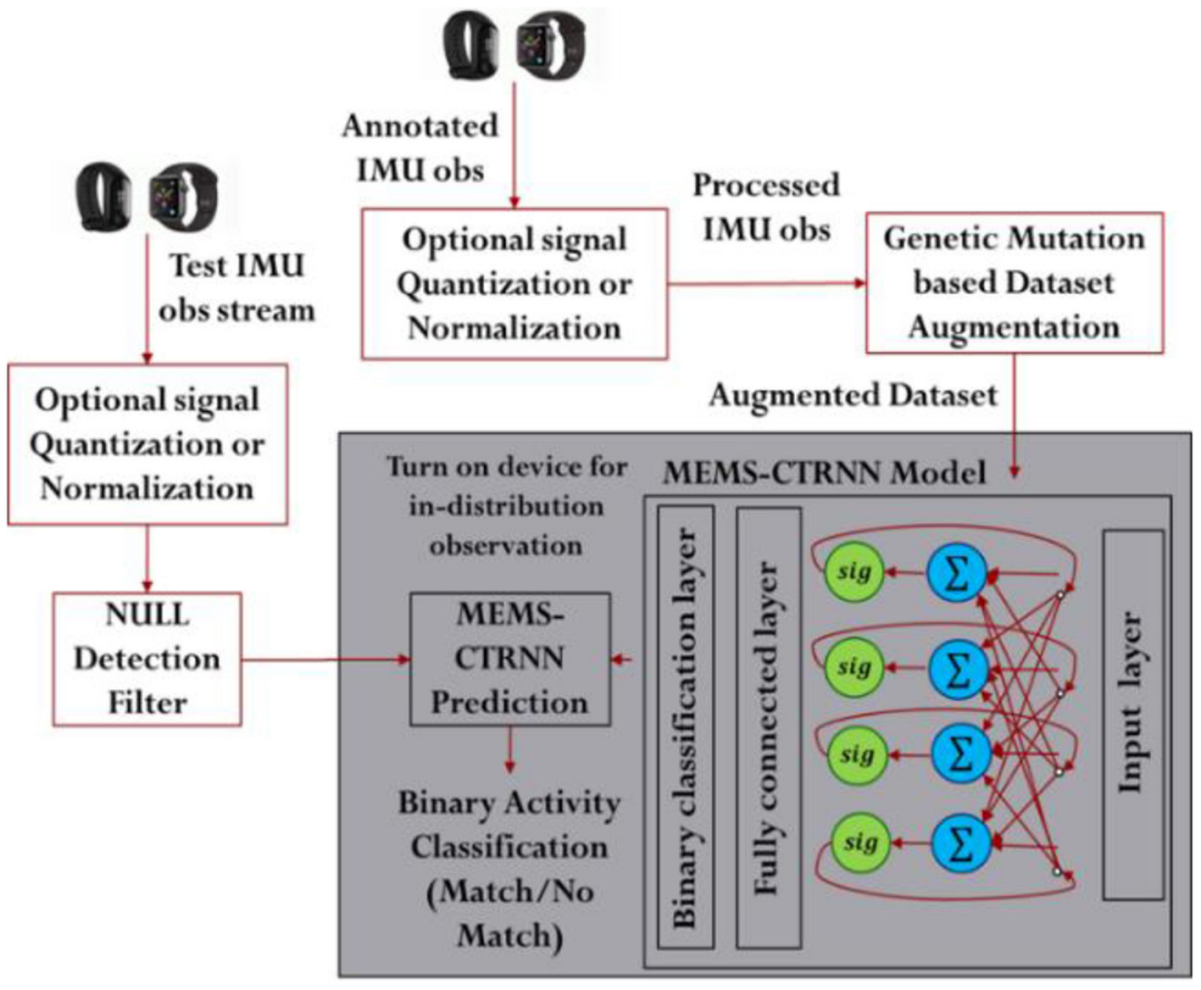
The HAR classification framework.

The objective of the chosen HAR application is to detect a given activity from a continuous sequence of motion acceleration sensor observations. The proposed learning architecture performs this detection by predicting whether the incoming sequence of observations belongs to an activity category or not. Thus, the HAR problem is converted into a binary classification problem, solved using the MEMS-CTRNN. In this binary classification problem, one class represents the target activity while the other class represents the null class or the remaining activities. The overall flow of the activity detection process using the proposed framework is illustrated in Figure 1. The framework has 3 major modules: (1) Input quantization module, (2) Genetic mutation-based training set augmentation, and (3) Training and testing module. Each of the elements in the flow chart is described in the subsequent subsections in detail.

### Input Quantization

Most wearable sensing systems seek to choose sensory data features that simplify the discrimination between different activities while being invariant across different executions of the same activity. Statistical measures, such as the data mean, median, and standard deviation are used as features for discrimination. These statistical measures often computed from data extracted from short frames of understanding of the application domain and the selection process of such features, have a significant impact on the classification performance of activity recognition systems. In this paper, we rely on a simpler, less computationally expensive approach of pre-processing known as signal quantization. This optional pre-processing step is used to improve the classification performance by utilizing a quantization strategy that normalizes and quantizes the sensor data to three levels (rise/fall/no change). We note that the quantization used here is applied to both the training and test data streams, as shown in Figure 1. Equation 2 outlines the quantization function for the incoming raw accelerometer signal *a* at time *t*. While quantization helps to simplify the problem for the underlying MEMS-CTRNN model, this is an optional step in the process flow as the physical implementation of the MEMS hardware for the case of simultaneous sensing and computing architecture may not allow quantization as will be explained in the next section. Here we adopt an input quantization that is based on the temporal rise and fall of the signal and is akin to feature extraction. Like a typical quantization, this quantization approach tends to preserve the signal trend without dealing with the exact values in the signal. Such extracted feature is much simpler for an RNN to learn when compared to an absolute input with infinite levels.


(2) $$\begin{array}{l} if\;a_{t}>a_{t+1}+\epsilon \;1\left(rise\right)\;\\ if\;a_{t}<a_{t+1}-\epsilon \;-1\left(fall\right)\;\\ if\;abs(a_{t+1}-a_{t})\leq \epsilon \;0\left(no\;change\right) \end{array}$$

### Genetic Mutation-Based Training Set Augmentation

The original training data is likely to be imbalanced. Thus, the purpose of introducing the genetic mutation module is to augment the original training dataset with more comprehensive data that can occur in the test data stream. The augmentation is only utilized during the training process to enrich and balance the training data and does not apply during the model testing or inference-making phase. We use a mutation genetic operator to synthesize a new observation from the original observation. A similar technique is used in (25) to generate automatic test data for software testing. Our approach is unique as we have control over two attributes of the resultant dataset: (1) the spread of mutation in time and (2) the extent of mutation in the motion intensity. The full process of genetic mutation training set augmentation is listed in Algorithm 1 and its parameter selection are detailed below.

**Algorithm 1:** Algorithm of the genetic mutation based augmentation.

1. Initialize the training dataset obsT where each element is created by sliding a fixed-horizon window of length l over the 3-D training input time-series accelerationsq.
2. Initialize the labels label_**T**_ where each element corresponds to each window in obs_T_. Set an element to “activity of interest” if input ground-truth_**T**_ indicates that more than 75% of observations in a window belong to the given input activity. Otherwise set the elemet to “null class”.
3. For each window in obsT for which corresponding label_**T**_ equals “activity of interest”, we select K random indices within the window and mutate the quantized acceleration level to a randomly chosen neighboring quantized level. The extent to which how far the quantization level can be randomly chosen is determined by M. We add the resultant K mutated window mut-window{1,…,K} to the training set obs_**T**_.
4. We label the corresponding elements in label_**T**_ for the newly added mut-window{1,…,K} as “activity of interest”.

**Figure 2:**
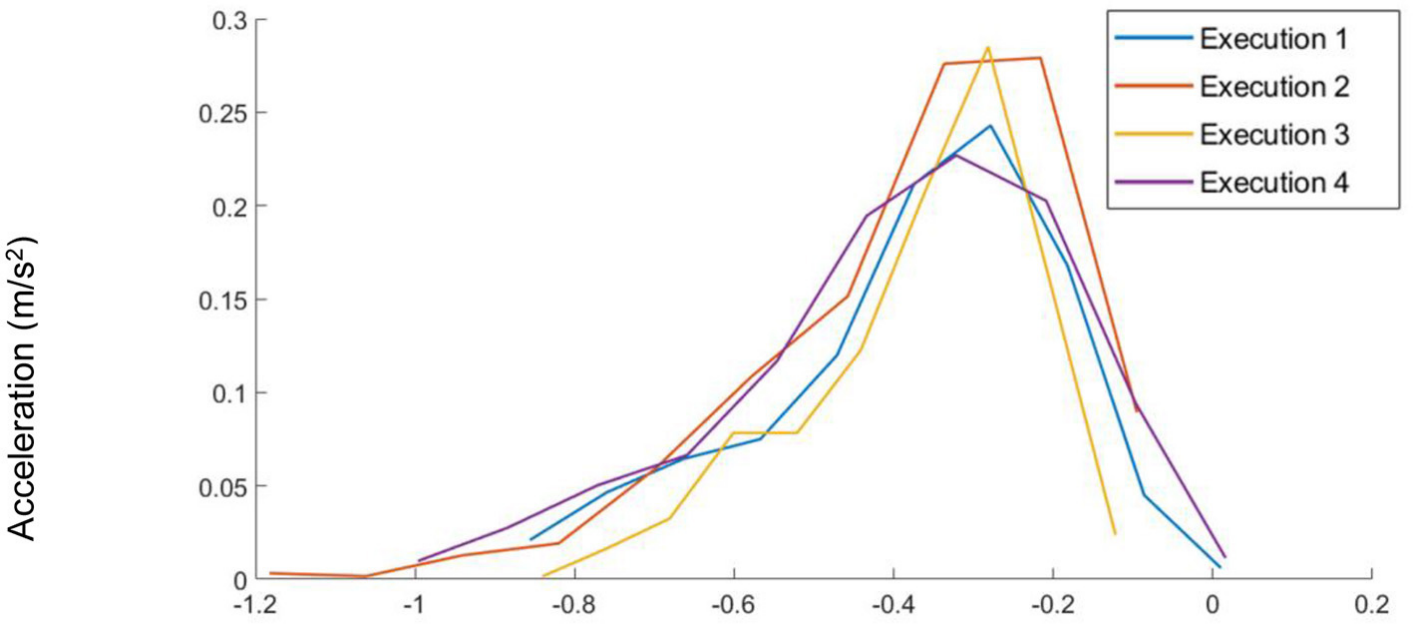
The distributions for non-normalized raw input data from the Y-axis Accelerometer for multiple executions of the same activity by the same subject. In this case, the variance is reasonably low.

The value of **M** depends upon the following factors:

- Large values of **M** may result in NULL class activity windows being incorrectly labeled as *activities of interest*.
- A very small value of **M** may cause us to miss some possible variation of activity by labeling the part of the *activity of interest* class. For example, because of a mutation, if the mutated acceleration value differs slightly from the original value, such little mutation is not enough to train the RNN for the inherent variations present whenever an activity is performed by the same subject but at different times. As an example of such variation, Figure 2 shows different executions of the same activity by the same subject.

The value of **K** depends upon the following factors:

- A large value of **K** will not preserve the motion features and may result in NULL class activity windows being incorrectly labeled as *activities of interest*.
- A small value of **K** may not bring the desired level of variation to the activity thus failing to capture some possible variations/nuances of the activity of interest. It may also lead to overfitting because the mutated data will look nearly identical to other non-mutated data points.
- It may also be noted that mutated windows **mut-window {1,…, K}** are assumed to represent a different execution of the same activity by the same subject or another subject.

### MEMS-CTRNN Model Architecture

In a previous work (26), we have shown that computing is possible using MEMS devices by approximating the qualitative nonlinear dynamics of CTRNNs. Mainly, MEMS device dynamics must include (1) Nonlinear instability to facilitate a distinct response change due to MEMS activation, and (2) memory, which is facilitated by hysteresis. Both behaviors are observed in electrostatic MEMS devices of different kinds. For example, an electrostatically actuated MEMS device exhibits a nonlinear response change at pull-in, when the excitation voltage results in an electrostatic force that exceeds the restorative force of the MEMS structure, causing the MEMS structure to contact the stationary excitation electrode. Hysteresis is inherent in the pull-in/release regime due to the nonlinearity of electrostatic forcing.

Alternative means of employing MEMS devices for CTRNN include using MEMS structures with nonlinear geometries, such as MEMS arches, and using internal feedback by using electrical resonance in low parasitic capacitance MEMS devices. For simplicity, this work focuses on using electrostatic MEMS devices, operated in the pull-in/release regime (5) to construct a MEMS CTRNN.

In the MEMS CTRNN pull-in/release implementation, we approximate the response of each electrostatically actuated MEMS device in the network as a single-degree of freedom spring-mass-damper system governed by (3), assuming that the influence of the MEMS inertia is very small compared to the influence of the damping on the MEMS response.:


(3) $$\begin{array}{l} \;\frac{2\zeta }{\omega_{n}}{\dot{z}}_{i}\left(t\right)+z_{i}\left(t\right)\\ =\frac{\varepsilon A}{2k{\left(d_{i}-z_{i}\left(t\right)\right)}^{2}}{\left(\sum_{j=1}^{N}w_{ij}V_{j}U\left(z_{j}\left(t\right)-d_{j}\right)+\theta_{i}\right)}^{2}\\ -\;\sum_{k=1}^{O}w_{in,k}\frac{1}{\omega_{n}^{2}}+I\left(t\right) \end{array}$$

where *z*_*i*_(*t*) is the state (the relative displacement between the MEMS moving proof mass and the fixed electrostatic electrode) of the *i*^*th*^ MEMS-CTRNN neuron at time t, ζ is the damping ratio, ω_*n*_ is the MEMS natural frequency, *w*_*ij*_ is the connection weight from the *j*^*th*^ neuron to *i*^*th*^ neuron, θ_*i*_ is a biasing signal applied to the ith neuron, *A* is the overlapping MEMS electrode surface areas, *k* is the MEMS linear stiffness, ε is the emissivity of air, *I*(*t*) is the input signal and for the case of simultaneous sensing and computing architecture (5) will be the actual acceleration signal (ý), otherwise, it will be an electrical signal (26), and *w*_*in, k*_ are the input weights, applied only to the MEMS devices in the input layer, and *V*_*j*_ is the total voltage signal on the *j*^*th*^neuron as defined by (4), computed recursively in simulation and updated automatically in neuromorphic applications:


(4) $$V_{j}=\sum_{k=1}^{N}w_{jk}V_{k}U\left(z_{k}\left(t\right)-d_{k}\right)+\theta_{j\;}\ldots$$

*U*(*z*_*k*_(*t*) − *d*_*k*_) is a unit step function, which is equal to 1 when *z*_*k*_(*t*) ≥ *d*_*k*_ and 0 otherwise. Here, *d*_*k*_ is the total electrostatic separation between the MEMS proof mass and the fixed electrode. This value is set to 42 × 10^−6^ in this paper and the rest of the MEMS parameters are listed in (5) and are chosen to operate the MEMS devices near Pull-in/Pull-out hysteresis to achieve the needed bistability for computing.

The proposed MEMS-CTRNN model consists of a fully connected input layer of MEMS input neurons to match the sensor inputs. If those neurons are optimized to sense acceleration, then the network can perform simultaneous sensing and computing (5). The second layer consists of several computing MEMS-CTRNN neurons. The number of MEMS-CTRNN neurons is kept low to orient the architecture toward, resource-constrained platforms and simplify their future physical implementation. This layer is followed by a fully connected output layer. The last layer is an output layer that provides binary classification decisions.

We used a discretized form of equation 1 in the training of our MEMS-CTRNN model. The training of the MEMS-CTRNN network is carried out by a custom MATLAB backpropagation-based framework. The framework was adapted from the framework proposed in (27). The base framework did not have MEMS-CTRNN nodes implemented, thus we resorted to implement and integrate these nodes into the framework. Additionally, the base framework did not support our novel genetic algorithm-based dataset augmentation and input quantization capabilities. MEMS-CTRNN also requires the integration of voltage *V*_*i*_ as listed in (4). This is the total voltage signal on each of the MEMS-CTRNN neurons. The voltage is a function of the output voltage of preceding neurons along with other parameters. The concept of voltage is novel and demanded extensive changes and testing for the framework code.

## Performance Evaluation

### Dataset

**Figure 3:**
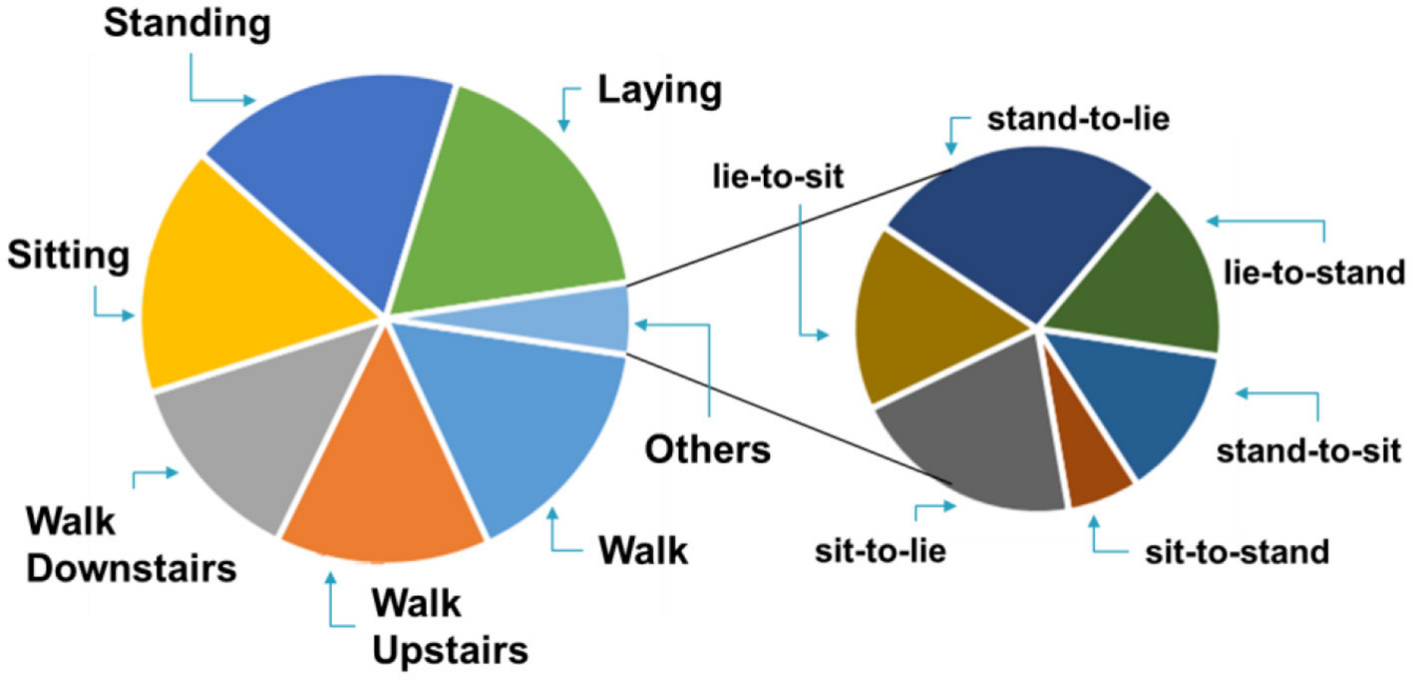
HAPT dataset activity proportion chart.

A publicly available and annotated Human Activity Recognition Dataset (HAPT) (28) was used to evaluate the proposed approach. The HAPT dataset contains time-domain signals that were captured at a constant rate of 50 Hz. The data was collected from 30 participants wearing a smartphone (Samsung Galaxy S II) on the waist during the experiment execution. The age range of participants varies from 19 to 48 years. The datasets include six basic types of activity i.e., WALKING, WALKING_UPSTAIRS, WALKING_DOWNSTAIRS, SITTING, STANDING, LAYING. The dataset also contains six postural transitioning activities i.e., stand-to-sit, sit-to-stand, sit-to-lie, lie-to-sit, stand-to-lie and lie-to-stand. Data related to one dynamic activity (walking) and four postural transitions (sit-to-stand, stand-to-sit, sit-to-lie, lie-to-sit) are considered for our experiment. Observations belonging to other activities also exist in the dataset, however, in this work, they are classified as null space observations. We randomly chose 70% of the data for the training and 30% as the test for our machine learning algorithm. Inputs to our algorithm are observation windows. Inputs to our algorithm were quantized according to equation (2). For each activity, best results were attained by setting the value for ϵ empirically to values close to the optimal value of ϵ ≈(**μ**_**a**_+**σ**_**a**_/**2**) available in the literature (29), where **μ**_**a**_ and **σ**_**a**_ are the mean and variance for the input distribution. The chosen training data was further augmented, as discussed in the preceding section, to balance the data. For training and testing, we consider each window to be a single sample. The proportions of activity samples in the dataset are shown in Figure 3. The walking activity observations make up 15.8% of the dataset while stand-to-sit, sit-to-stand, sit-to-lie, and lie-to-sit constitute 0.6, 0.3, 1.0, and 0.8% of the overall dataset, respectively.

### Activity Detection Performance Using Traditional CTRNN and LSTM Models

To demonstrate the effectiveness of the proposed MEMS-CTRNN model, we first compare the performance of traditional CTRNNs and Long Short-Term Memory (LSTM) networks as a baseline. Next, we compare the performance of our MEMS-CTRNN approximation to our baseline models. LSTM is chosen as a commonly used architecture to perform human activity classification. We use the well-cited deep forward LSTM model proposed in (30), which contains multiple layers of recurrent units that are connected “forward” in time. The model architecture is simple yet powerful enough to produce reliable HAR results over publicly available HAR Datasets. To create a CTRNN model, we simply replace the LSTM nodes with the CTRNN nodes.

#### Evaluation of Performance With Accelerometer and Gyroscope Inputs

To establish a baseline, we first present the performance results for traditional CTRNN, and LSTM based activity detection in Table 2. Both models contain a single hidden layer of 6 recurrent neurons. There are also 6 input neurons to match the number of the input sensors (3D accelerometer and 3D gyro) and 2 output statuses (Activity Detected, Not detected) for each model. It is worth mentioning that we have obtained very high classification accuracy using another common CTRNN and LSTM topology that assigns an input neuron for each observation in the activity period (window). However, we chose not to use this topology as it will result in having many neurons in the network input layer, which may create a physical implementation challenge for the MEMS CTRNN. During the training phase, each observation window is labeled to belong to the activity of interest class, whenever 75% or more observations in each sliding window belong to the associated activity. Otherwise, the window is associated with the Null class. Thus, the results in table 2 use the 75% threshold while labeling the window. Also, the inputs to the models were quantized into 3 levels as described in section Input Quantization. We observe that even with a limited number of neurons, the performance results for the LSTM and CTRNN models have the lowest of 89 and as high as 96% activity detection accuracy.

**TABLE 2:** Baseline performance results for CTRNN and LSTM activity detection models for 5 subjects and 5 activities.

**Activity name**	**Average accuracy for 6 neurons hidden layer model for quantized accelerometer** **+** **Gyro Inputs. (75% labeling threshold)**	
	**LSTM**	**CTRNN**
Walk	96.4%	89.2%
Sit-to-Stand	94.8%	88.8%
Stand-to-Sit	93.9%	90.2%
Sit-to-Lie	95.3%	89.1%
Lie-to-Sit	94.0%	88.9%

#### Evaluation of Performance With Only Accelerometer Inputs

Next, we investigate the impact of the removal of gyroscope inputs on the accuracy of LSTM and CTRNN models. In this scenario, we only consider the quantized 3D accelerometer inputs to the model, thus we have only three input neurons. Aside from the removal of the 3-axis gyroscope data and reducing the number of the input neurons, the model configurations were kept consistent with the model described in section Evaluation of performance with accelerometer and gyroscope inputs. Table 3 lists the accuracy results for the reduced input models. The table shows that removing the gyroscope inputs has a minimal impact on the model's accuracy. This finding may have a substantial impact on reducing the complexity of implementing the MEMS CTRNN hardware as there is no need to fabricate and integrate gyroscopes in the computing hardware.

**TABLE 3:** Baseline performance results (without Gyro inputs) for CTRNN and LSTM activity detection models for 5 subjects and 5 activities.

**Activity name**	**Average accuracy for 6 neurons hidden layer model for quantized accelerometer inputs (75% labeling threshold)**	
	**LSTM**	**CTRNN**
Walk	93.8%	86.3%
Sit-to-Stand	91.6%	86.8%
Stand-to-Sit	90.7%	87.0%
Sit-to-Lie	92.4%	87.2%
Lie-to-Sit	91.9%	86.7%

### Activity Detection Performance for MEMS-CTRNN Model While Accounting for Its Physical Implementation Limitations

In this section, we evaluate the MEMS-CTRNN model performance for the binary human activity classification task. Again, our goal here is not to outperform any existing HAR technique but rather to show that the proposed MEMS-CTRNN method produces comparable performance to commonly cited CTRNN and LSTM based activity classification models (3, 8) with the advantage of consuming less power. Table 4 shows the classification accuracies for the MEMS-CTRNN simulation model with different scenarios. For example, the cell in the first row and second column in the table shows that the MEMS-CTRNN implementation (with quantized acceleration inputs) has an accuracy that is comparable to the traditional CTRNN implementation reported in Table 3 when using only accelerometers inputs. For practical hardware implementation of MEMS-CTRNNs, however, some limitations may exist, especially for the simultaneous sensing and computing architecture, to attain maximum power reduction. For example, input quantization may not be possible for the sensing and computing MEMS architecture as in such architecture the MEMS that perform computing in the input layer will sense the acceleration signal directly in an analog fashion (5). Table 5 shows an accuracy drop only by around 5% when non-quantized inputs are used to train and validate the MEMS-CTRNN model, similar to previously reported showcases of traditional RNNs (22, 23).

**TABLE 4:** Average accuracy observations for MEMS-CTRNN models for varying input dimension sizes and input quantization conditions.

**Accelerometer axis**	**Non-Quantized accelerometer inputs**	**Quantized accelerometer inputs**
	**Model accuracy for Stand-to-sit activity (5 subjects)**	**Model accuracy for Stand-to-sit activity (5 subjects)**
X. Y, Z	78.2%	83.5%
X, Y	51.9%	55.3%
X, Z	55.4%	58.5%
Y, Z	56.9%	59.7%

**TABLE 5:** Impact of changing labeling threshold.

**Activity name**	**Average accuracy for 16 neurons hidden layer model**			**Average accuracy 16 neuron hidden layer model**		
	**(50% labeling threshold)**			**(75% labeling threshold)**		
	**Non-Quantized accelerations**			**Non-Quantized accelerations**		
	**LSTM**	**CTRNN**	**MEMS-CTRNN**	**LSTM**	**CTRNN**	**MEMS-CTRNN**
Walk	78.4%	68.3%	66.9%	87.0%	78.7%	76.5%
Sit-to-Stand	75.9%	62.1%	58.6%	85.3%	77.9%	77.6%
Stand-to-Sit	72.7%	59.4%	57.5%	84.4%	79.5%	78.9%
Sit-to-Lie	73.2%	62.2%	58.7%	87.1%	78.4%	78.7%
Lie-to-Sit	72.4%	60.6%	58.2%	86.5%	77.9%	78.0%

Another aspect of the implementation limitation of the MEMS-CTRNN simultaneous sensing and computing architecture worth exploring is the dimensions of input acceleration. While 3-D accelerometers are commercially available, we expect optimizing its design parameters to perform simultaneous sensing and computing purposes is more challenging than our recent implementation attempts for lower input dimension MEMS CTRNN (5). We tested the accuracy of our proposed model for 2 axis accelerometer input (all possible combinations). Rows 2–4 in Table 4 demonstrate the impact of using the non-quantized compared to quantized inputs for all possible combinations of accelerometer inputs. Accuracy readings are tabulated for the proposed MEMS-CTRNN model. The result in the table shows that reducing the accelerometer dimensions will result in a significant reduction in the classification accuracy.

**Figure 4:**
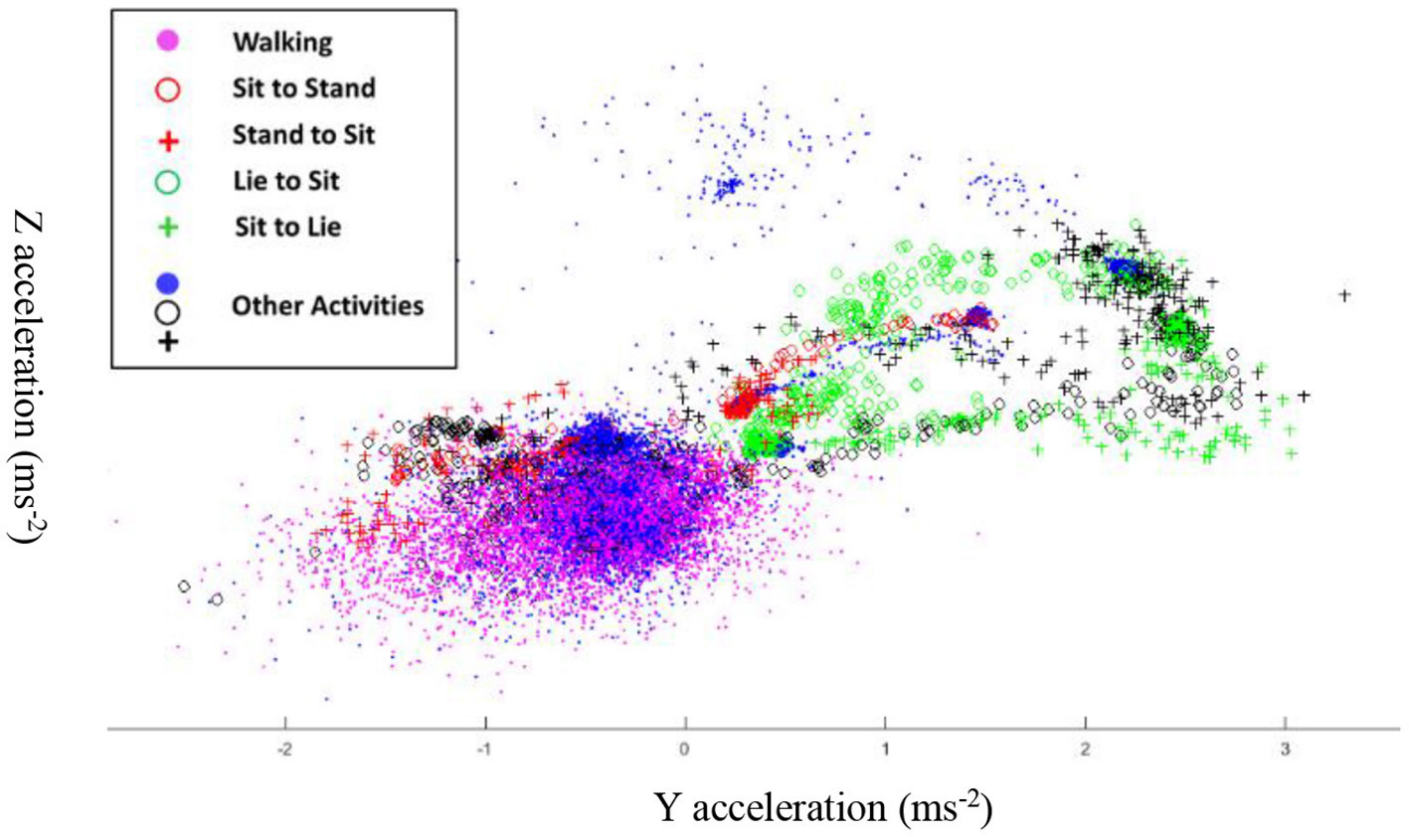
Clusters of activity observations on the YZ plane.

To explore the effect of the number of acceleration dimensions on the classification accuracy drop, we visualize the distribution of each activity observation in the 2-D space in Figure 4. In this figure, we chose the 2-D YZ as an example. The figure shows that the observations are forming complex clusters in the YZ plane with overlapping observations belonging to different activities. The above observation explains the significant decrease in accuracy when we reduce the input dimensions to our learning model. The reduced dimensions accuracy analysis presented in Table 4 helps establish the tradeoff between MEMS fabrication challenges and classification accuracy using this architecture. However, we reiterate here that, first while challenging, it is still possible to fabricate an optimized 3-D accelerometer for the simultaneous sensing and computing MEMS CTRNN architecture, which is beyond the scope of this paper. Second, for 3-D and higher dimension input applications, one can always utilize the MEMS CTRNN only computing architecture (26), which separates between the actual sensor layer and the MEMS CTRNN input neuron layer like any other traditional machine learning algorithm, with the additional complexity of sensor interfacing and conditioning circuit.

### MEMS-CTRNN Model Accuracy Sensitivity Analysis to Threshold Value, Network Size, and Time Constant

In this section, we present a thorough analysis of some of the factors that can significantly impact MEMS-CTRNN accuracy. For comparison purposes, when applicable, we also present the impact of those parameters on the traditional LTSM and traditional CTRNN.

#### Impact of Network Size on Model Accuracy

**Figure 5:**
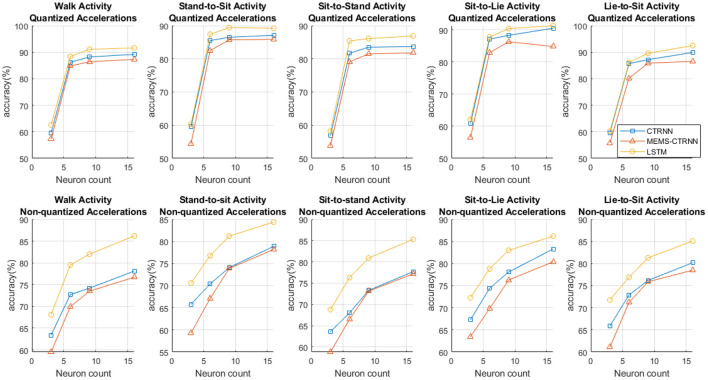
The accuracy charts for LSTM, CTRNN, and MEMS-CTRNN across 5 activities and 5 subjects. The X-axis represents the number of neurons in the hidden layer (3, 6, 9, 16).

We trained the MEMS-CTRNN model with a varying number of neurons in the hidden layer (3, 6, 9, 16) and compared the observed accuracy with LSTM and CTRNN models. The resulting comparison is shown in Figure 5. We chose the numbers of neurons to not exceed 16 as such larger coupled MEMS networks may be impractical to fabricate. The figure shows that, for quantized inputs, the performance of all algorithms is nearly saturated when >6 neurons are used. We note that the performance of the LTSM network is better than CTRNNs in Non-quantized data applications (bottom row of Figure 5), compared to applications with quantized data (top row of Figure 5). Furthermore, the performance does not saturate when more than 6 neurons are used.

#### Impact of Changing Labeling Threshold During the Training Process

During the genetic mutation-based training process (Section Genetic Mutation-based Training Set Augmentation), the observations window is labeled to belong to the *activity of interest* class whenever 75% or more observations in each sliding window belong to the associated activity. Ideally, this threshold should be chosen to be higher, but this may increase the false positive rate if the activity of interest is not spread evenly across the observation windows. On the other hand, we observed that dropping the threshold significantly reduces the model accuracy. In Table 5, for example, we observe that overall activity detection accuracy drops by an average of 14.6% even for a network of 16 neurons when a threshold value of 50% is used. The underlying hypothesis behind this result can be that labeling windows with many observations, that do not belong to the activity of interest class, introduce challenges for the MEMS-CTRNN neurons to be able to classify complex clusters formed by the new low threshold.

#### Impact of Varying CTRNN Time-Constant on Model Accuracy

A CTRNN consists of continuous-time neurons. Each of these neurons is modeled by a linear first-order differential equation and its time scale is determined by the time constant τ. where τ is a characteristic of the mechanical design of the MEMS node (damping ratio and MEMS natural frequency) given by:


(5) $$\begin{array}{r} \tau =2\zeta /\omega_{\text{n}}\ldots \end{array}$$

A CTRNN neuron with a small τ experiences a strongly decaying behavior. It also reacts rapidly and nearly instantaneously to the current inputs. Contrarily, a large τ leads to slowly varying states, as the neuron will maintain its previous internal state and sluggishly react to current inputs. Each neuron in CTRNN can have a unique value for τ. However, for simplicity in our MEMS-CTRNN simulation, we consider the same constant value of τ=*0.0017* seconds for all MEMS nodes in the network. Next, we investigate the effect of varying τ, by varying the value of ω_*n*_ by a multiplier constant *P* with the following set of values:


(6) $$\begin{array}{r} \text{P}=\left\{\frac{1}{5},\frac{1}{4},\frac{1}{3},\frac{1}{2},1,2,\;3,\;4,\;5,\;6,\;7,\;8,9,10\right\}\ldots \end{array}$$

**Figure 6:**
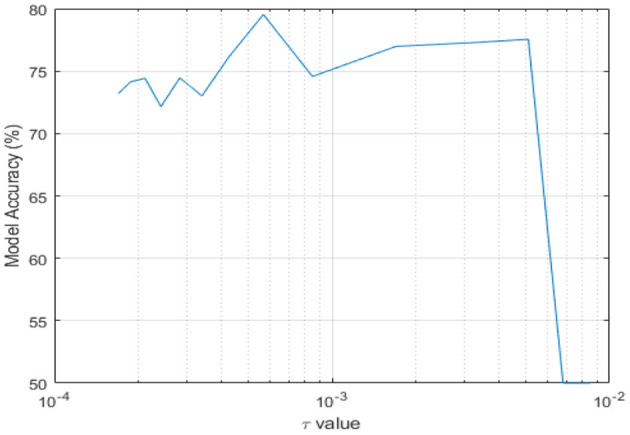
Impact of the MEMS time constant on the classification accuracy in a MEMS-CTRNN.

These values are chosen based on the constraints dictated by the MEMS physical design characteristics. For all possible values of τ, given the multiplier values, we evaluated the activity recognition performance. Figure 6 illustrates the impact of the τ values on the accuracy of a MEMS-CTRNN model for stand-to-sit activity with non-quantized accelerations and a 75% labeling threshold. Literature (31) frequently uses values of τ to learn patterns that exist over multiple time scales in a time series. This is particularly helpful for motion sensor-based time-series data as well. Therefore, we use the time constant τ as a hyperparameter in our network to study its effects on the accuracy of MEMS classification. We observe from Figure 6 that the accuracy peaks for τ = 0.000566 seconds which corresponds to a timescale that the MEM-CTRNN responds to most favorably. The accuracy drops to 50% when the values of τ approach 0.0068 seconds. The accuracy continues its downward trend as the values of τ increase beyond 2e-2, which corresponds to the MEMS-CTRNN losing its memory and reacting only to current inputs. It is worth mentioning that in the above simulation, we still use a constant value for this τ parameter for all the MEMS CTRNN nodes. This only enables the MEMS CTRNN to detect patterns at a single “average time-scale.” As future work, we plan to train a different τ for each neuron in the network so the network can learn the patterns that exist on multiple time scales. As reported for traditional CTRNN (31), we expect that this approach will enhance the accuracy of our framework by a significant margin, in contrast to the currently presented approach, which detects only the “dominant pattern” in the time-series signal.

## Conclusion

In conclusion, we demonstrated the adaption of a traditional machine learning architecture to train a network of MEMS devices to simulate the response of MEMS-based CTRNNs in HAR applications. The main advantage of this MEMS-based CTRNN is to reduce the computational cost through the inherent dynamics of the MEMS devices, with the added advantage of optionally performing some computation at the sensor level. The MEMS-CTRNN model discussed in this paper is not a one-to-one emulation of CTRNNs in-*silico*. However, this work shows that the use of MEMS devices to emulate CTRNNs yields nearly identical results, due to the previously demonstrated qualitative similarities of response between MEMS-CTRNNs and CTRNNs. In this work, the performance of the MEMS-CTRNN has been shown both using input pre-processing through input quantization and without the pre-processing. When input pre-processing is available, the MEMS-CTRNN can perform HAR with high accuracy. Input pre-processing may still be foregone with a 5% loss of accuracy.

This work further investigated the influence of parameters such as network size, input quantization level, and MEMS-CTRNN time constant τ on the HAR task performance. We show that the performance of MEMS-CTRNN plateaus when implementing input quantization as the number of neurons increases. This behavior is also observed in traditional CTRNNs and LSTMs. This behavior differs when input quantization is not used. We also show that the input-window labeling threshold is a key parameter in the training process, which may significantly reduce the classification accuracy if chosen poorly. A threshold of 75% was shown to be appropriate for binary classification HAR application. Finally, the choice of MEMS-CTRNN time constant was shown to also be of paramount importance. In general, for our application, a small-time constant (below 8 × 10^−3^) was shown to render the MEMS-CTRNN memoryless, resulting in a rapidly deteriorating response.

Overall, the response of the MEMS-CTRNN was shown to be acceptable compared to state-of-the-art architectures in HAR applications. We reiterate here that the goal of this paper is not to outperform LSTM. Rather, this work provides a low-power neuromorphic alternative that may be better suited for computationally constrained devices. The performance of the MEMS-CTRNN may be improved by training the MEMS-CTRNN time constant to react to input signals with various timescales to enable the extraction of further information of time-series signals.

## Code Availability

The MEMS-CTRNN code can be accessed in the author repository^1^.

## Data Availability Statement

Publicly available datasets were analyzed in this study. This data can be found here: https://archive.ics.uci.edu/ml/datasets/human+.

## Author Contributions

FA, MH, and RJ: conceptualization. FA, MH, and ME-U-D: methodology. ME-U-D and FA: software and writing—original draft preparation. SP, MH, and RJ: writing—review and editing. FA, SP, and RJ: project administration. FA, RJ, SP, and FA: funding acquisition. All authors have read and agreed to the published version of the manuscript.

## Funding

This research was funded by the National Science Foundation, Grant Number (#1935598, #1935641).

## Conflict of Interest

The authors declare that the research was conducted in the absence of any commercial or financial relationships that could be construed as a potential conflict of interest.

## Publisher's Note

All claims expressed in this article are solely those of the authors and do not necessarily represent those of their affiliated organizations, or those of the publisher, the editors and the reviewers. Any product that may be evaluated in this article, or claim that may be made by its manufacturer, is not guaranteed or endorsed by the publisher.

## Footnotes

^1^Available online at: https://github.com/em22ad/CTRNN_timeseries/tree/main/MatDL
